# Septin Organization and Functions in Budding Yeast

**DOI:** 10.3389/fcell.2016.00123

**Published:** 2016-11-03

**Authors:** Oliver Glomb, Thomas Gronemeyer

**Affiliations:** Department of Molecular Genetics and Cell Biology, Ulm UniversityUlm, Germany

**Keywords:** septins, budding yeast, GTPase, crystal structure, cytokinesis

## Abstract

The septins are a conserved family of GTP-binding proteins present in all eukaryotic cells except plants. They were originally discovered in the baker's yeast *Saccharomyces cerevisiae* that serves until today as an important model organism for septin research. In yeast, the septins assemble into a highly ordered array of filaments at the mother bud neck. The septins are regulators of spatial compartmentalization in yeast and act as key players in cytokinesis. This minireview summarizes the recent findings about structural features and cell biology of the yeast septins.

The classical Hartwell screen revealed genes involved in cell cycle regulation of the budding yeast *Saccharomyces cerevisiae* (Hartwell, 1971). Beside many others, the screen showed mutations in the four genes CDC3, CDC10, CDC11, and CDC12 (**c**ell **d**ivision **c**ycle). These were later classified as members of the septins, a new family of cytoskeletal proteins in the budding yeast (Sanders and Field, 1994; Pringle, 2008). Another mitotic septin, Shs1 (**s**eventh **h**omolog of **s**eptin), was identified more than two decades after the initial screen (Mino et al., 1998). In meiosis, Cdc11 and Cdc12 are replaced by two other septins, Spr28 and Spr3, respectively (Ozsarac et al., 1995; De Virgilio et al., 1996). These two septins are expressed under an own promoter and the resulting meiosis-specific septin complexes exhibit distinct functionalities than their mitotic counterparts (Garcia et al., 2016). In this review we will focus on the mitotic septins.

The mitotic yeast septin proteins can be recombinantly expressed and purified from *E. coli* (Bertin et al., 2008; Renz et al., 2013). Analysis of mutated or truncated recombinant septin preparations uncovered the organization of the individual subunits: They assemble into hetero-oligomers, called rods, with the order Cdc11-Cdc12-Cdc3-Cdc10-Cdc10-Cdc3-Cdc12-Cdc11 with Shs1 sometimes replacing the terminal subunit Cdc11 (Bertin et al., 2008; Garcia et al., 2011). These rods appear *in vitro* as short filaments of 32 nm length under high salt conditions (Kaplan et al., 2015). Septin rods can be induced to form long septin filaments *in vitro* by lowering the salt concentration of a buffer that otherwise keeps the rods stable in solution (Bertin et al., 2008; Renz et al., 2013). These filaments are about 1.5 μM long and exhibit a thickness within the range observed for microtubuli (Kaplan et al., 2015). Hexameric rods lacking the terminal subunit Cdc11 fail to form filaments suggesting that filament formation occurs end-over-end via the terminal subunit Cdc11 (Bertin et al., 2010). This observation was further fostered by applying single molecule localization microscopy to rods with their central and terminal subunits labeled with fluorescent dyes (Kaplan et al., 2015). Another study using septin preparations labeled with suitable FRET donor and acceptor dyes confirmed the end-over-end assembly via Cdc11 and showed that this interaction has a high affinity with a K_D_ in the nano-molar range (Booth et al., 2015). Alternative modes for filament formation via other subunit contacts could be excluded by evaluation of different combinations of subunits labeled with the FRET compatible dyes (Booth et al., 2015).

Septin octamers containing Shs1 instead of Cdc11 as terminal subunit do not form linear filaments *in vitro* but rather curved bundles that assemble into closed rings (Garcia et al., 2011) suggesting that Shs1 is necessary to form also curved structures *in vivo*. Recombinant septin rods exclusively capped with Shs1 do not assemble end-over-end into filaments whereas Shs1-Cdc11 contacts are formed *in vitro* (Booth et al., 2015).

Crystal structures of the human subunits SEPT2 and SEPT7 provided invaluable insight into the architecture of subunit contacts and filament formation (Sirajuddin et al., 2007, 2009; Zent et al., 2011). Structurally, septins are GTP-binding proteins sharing similarities with the small GTPases of the Ras family (Leipe et al., 2002). A central conserved G-domain is flanked by variable N- and C-terminal extensions. The G-domain core resembles the conserved Ras structure with six β-strands and five α-helices. Structural elements from Ras like the switch I and switch II motifs and the P-loop (Schweins and Wittinghofer, 1994) classify the septins as *bona fide* GTPases. Besides these canonical structural elements, the septin G-domain harbors a septin unique element at its C-terminus that forms a distinct α-helix, α6 following the domain nomenclature from Ras, which points away from the G-domain in a 90° angle. Other septin specific features are the presence of a short N-terminal helix, α0, and a distinct loop with two antiparallel strands β7 and β8.

The crystal structure of a septin filament consisting of SEPT7, SEPT6, and SEPT2 revealed the nature of the binding interface of the septin subunits within the filament (Sirajuddin et al., 2007): Interactions between two adjacent G-domains (called G-interface) alternate with interactions of two adjacent N- and C-termini of two subunits (called NC-interface). The filament subunit is a hexamer with the order SEPT7-SEPT6-SEPT2-SEPT2-SEPT6-SEPT7. Here SEPT7 forms a G-interace with SEPT6, SEPT6, and SEPT2 form a NC-interface, SEPT2 and SEPT2 a G-interface and so on. The α6 helix is the prominent element of the NC-interface. C-terminal extensions like predicted coiled-coils do not seem to contribute to the NC-interface (Sirajuddin et al., 2007). The G-interface is composed of a complex network of interactions between different amino acid residues of the respective opposite subunit. Especially the interacting Val, His, and Trp residues located in the β7 and β8 sheets are highly conserved among human and fungal septins. Based on the high sequence homology between fungal and human septins one would assume that the architecture of the G-interface is shaped similarly in yeast septins. However, in the recently solved crytsal structure of Cdc11 (Brausemann et al., 2016, Figure 1), the terminal subunit of the *S. cerevisiae* septin rod, the loop containing the β7 and β8 sheets is not structured and the base of the loop points away from the presumed G-interface in an approximetaly 90° angle (Figure 1). This, together with the finding that Cdc11 dimers are maintained via the C-terminal coiled-coil extensions of the Cdc11 monomers and not via a NC- or G-interface, suggests that at least some yeast septins have interaction interfaces that might differ from the “classic” NC- and G-interfaces from the human septins.

**Figure 1:**
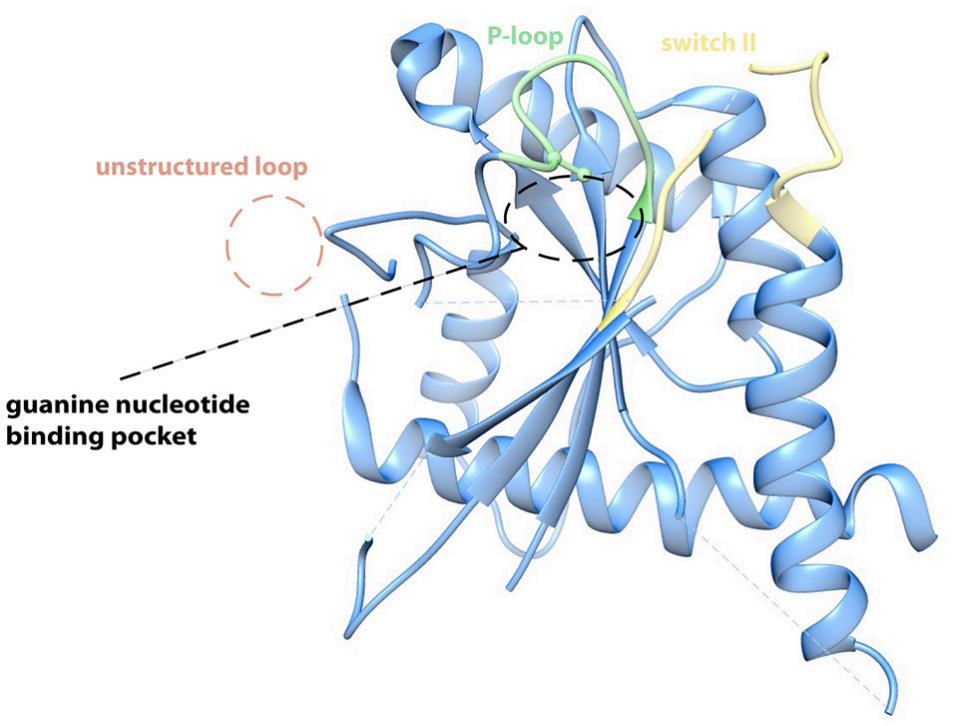
**Crystal structure of Cdc11_**20−298**_ (PDB-ID 5AR1)**. The protein crystallized in its apo form. The P-loop, the switch II loop and the anti-parallel ß7–ß8 sheets contribute to the G-binding interface in the human SEPT2 and SEPT7 (Sirajuddin et al., 2007; Zent et al., 2011). In the Cdc11 crystal the ß7–ß8 hairpin is unstructured and points away from the binding interface in a 90° angle, whereas P-loop (green) and switch II region (yellow) show high similarities. An arginine within the P-loop of Cdc11 possibly hinders effective binding of a nucleotide and thereby possibly explains why Cdc11 is incapable of binding a nucleotide (Brausemann et al., 2016). Figure created with Chimera vers. 1.10.2. from PDB structure 5AR1.

Cdc11 exhibits one more unique feature: The crystal structure is from the apo form and does consequently not contain a nucleotide (Brausemann et al., 2016). All yeast and human septins harbor an absolute conserved lysine residue in the canonical P-loop sequence GXXXXGKS/T. The P-loop is usually in contact with the β-phosphate of the bound GTP or GDP. Instead of the conserved lysine, Cdc11 has an arginine in this position of the P-loop whose side chain would collide with the β-phosphate of any bound nucleotide (Figure 1). Is Cdc11 not capable of binding a nucleotide? This assumption is fostered by the finding that cells expressing *cdc11* alleles that contain mutants in the P-loop show wild type like phenotype at 30°C (Casamayor and Snyder, 2003). Furthermore, only Cdc10 and Cdc12 have been shown to bind and hydrolyze GTP whereas GTPase activity could not be shown for Cdc3 and Cdc11 (Versele and Thorner, 2004).

In the living yeast cell, the septin filaments assemble at the bud neck in an organized array, the so-called septin ring. This ring undergoes different cell cycle dependent architectural transitions (Figure 2). In early G_1_-phase, the septins are recruited to and accumulate at the presumptive bud site in a patch-like structure. Shortly before bud emergence, the septins assemble in a Cdc42 dependent manner into a ring marking the future site of bud growth and cytokinesis (Gladfelter et al., 2002; Caviston et al., 2003; Iwase et al., 2006). This initial recruitment of the septins to the future bud site depends besides Cdc42 on its effectors Gic1 and Gic2, and the action of the cyclin-dependent kinases Cdc28 and Pho85 (Tang and Reed, 2002; Iwase et al., 2006; Egelhofer et al., 2008; Okada et al., 2013). Evaluation of the mechanisms underlying the annealing of recombinant septin rods and formation of filaments on lipid bilayers suggests that septin filaments are formed *in vivo* at the plasma membrane from small complexes that diffuse in two dimensions (Bridges et al., 2014). Initial recruitment to the plasma membrane is supposed to be mediated by phospholipids. In an *in vitro* lipid monolayer model PIP2 enhanced the filament assembly rate of septin hetero-octamers (Bertin et al., 2010).

**Figure 2:**
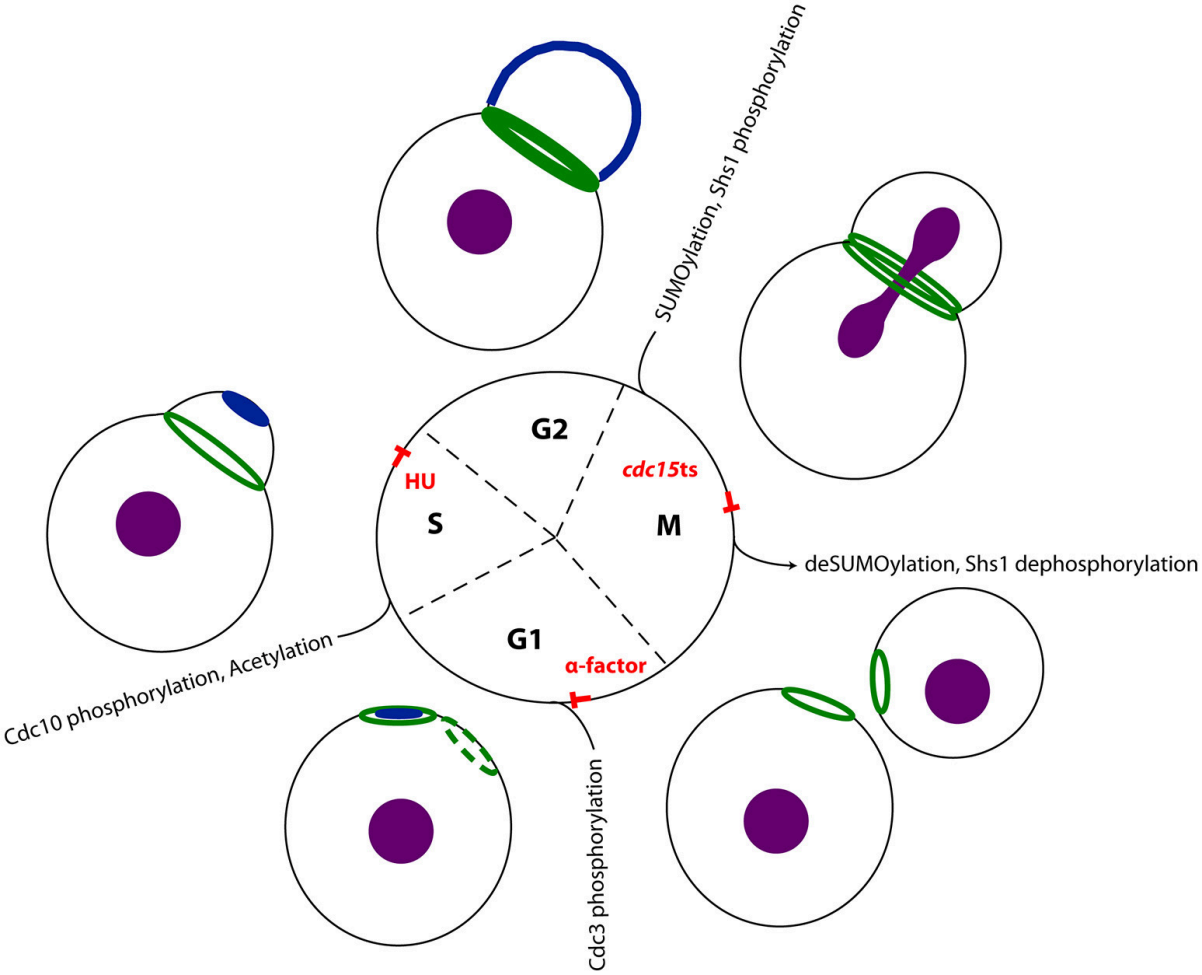
**Septins in the cell cycle of ***S. cerevisiae*****. The septins undergo cell cycle depending transitions: In G1 phase active Cdc42 (dark blue) polarizes at the plasma membrane, defining the presumptive bud site. The septins (dark green) are recruited to this site and remain as a ring at the neck upon bud emergence. This ring expands into a stable hourglass-shaped collar until the onset of mitosis. In late anaphase the septin collar splits into two distinct rings. In a recent study, Renz et al. detected interaction partners systematically at different stages of the cell cycle and could thereby reconstitute a time-resolved interactome of the septin rod (Renz et al., 2016). Treatment of the yeast cells with alpha factor arrested cells in early G1 phase, where no septin structure was visible. Using hydroxyurea arrested the cells in S-phase, where a stable septin ring was visible at the bud neck. Finally, a temperature shift in cells carrying a *cdc15-1* allele, allowed determining interaction partners with split septin rings. The respective cell cylce blocks and major known post translational modifications (Hernández-Rodríguez and Momany, 2012) of the respecive cell cycle state are indicated. The nucleus is colored in purple.

After bud formation, the septin ring expands into a stable hourglass-shaped collar that is present at the bud neck until the onset of mitosis (Vrabioiu and Mitchison, 2006; Oh and Bi, 2011; Ong et al., 2014). Before cytokinesis, the septin collar splits into two distinct rings, one located at the mother and one at the daughter site of the bud neck. The contractile actomyosin ring (AMR), which powers the ingression of the cleavage furrow and septum formation, is assembled between the two septin rings (Wloka and Bi, 2012). Myo1, one essential constituent of the AMR, is recruited to the site of cytokinesis via the septin interacting protein Bni5 (Fang et al., 2010; Schneider et al., 2013). Bni5 in turn associates with the C-terminal extensions of the septin subunits Cdc11 and Shs1 (Finnigan et al., 2015).

After completion of cell separation, the old septin rings are disassembled and septin subunits are partially replaced and recycled for the next round of the cell cycle (McMurray and Thorner, 2008).

Taken together, the septins act mainly as scaffold for other proteins that are recruited to the bud neck or the site of cytokinesis, respectively. Additionally, the septins function as a diffusion barrier for proteins and organelles at the cortex of the bud neck (Luedeke et al., 2005; Shcheprova et al., 2008; Caudron and Barral, 2009; Orlando et al., 2011).

The transitions that the septins undergo throughout the cell cycle are supposed to be regulated by posttranslational modifications: Transition of the septin ring into a stable septin collar after bud emergence is associated with the phosphorylation and acetylation of certain subunits (Mitchell et al., 2011; Hernández-Rodríguez and Momany, 2012). For example, the p21-activated kinase Cla4 phosphorylates Cdc10 after bud emergence and a deletion of *CLA4* strongly affects the timely formation of septin structures (Dobbelaere et al., 2003; Kadota et al., 2004; Versele and Thorner, 2004). The splitting of the septin collar at the onset of cytokinesis is supposed to be initiated by a collective switch in the orientation of the septin filaments from parallel to perpendicular to the growth axis of the cell (DeMay et al., 2011). The switch is accompanied by at least two different modifications. First, the bud neck kinase Gin4 phosphorylates Shs1 at residues different from those being modified in G_1_-phase (Mortensen et al., 2002). Gin4 is recruited together with the septins to the presumptive bud site, co-localizes with the septins at the bud neck for the complete cell cycle and disappears from the bud neck after splitting of the septin collar (Longtine et al., 1998; Mortensen et al., 2002; Au Yong et al., 2016). Second, the small ubiquitin-like modifier (SUMO) Smt3 is covalently attached to Cdc3, Cdc11, and Shs1 at the mother site of the bud (Johnson and Blobel, 1999). Phosphorylation events play apparently an important role in septin structure transitions and septin organization and several more kinases have been identified to interact with the septins: Gin4 (Dobbelaere et al., 2003), Kcc4 (Okuzaki and Nojima, 2001; Kozubowski et al., 2005), Ste20 (Ptacek et al., 2005), Hsl1 (Finnigan et al., 2016), Elm1 (Kang et al., 2016), and Kin2, the ortholog of animal MARK/PAR-1 kinase (Yuan et al., 2016), were all identified as septin interactors.

However, for most of these regulatory proteins the exact timing of the interaction with the septins remains unknown and one can speculate that much more interactions exist but are not identified so far. Recently, Renz et al., performed a systematic screen for septin interaction partners throughout the cell cycle by combining cell cycle synchronization at stages with a distinct septin structure (Figure 2) with quantitative mass spectrometry to timely resolve the septin interactome (Renz et al., 2016). Yeast cells were synchronized in G1 phase with alpha factor, in S phase with hydroxyurea and in late anaphase with the help of a tempertaure sensitive *cdc15* allele. This strategy allowed to link interaction partners to specific transition states of septin structures. Distinct sets of regulatory proteins were found to interact with the septins at certain stages of the cell cycle: Gin4 and the anillin-like protein Bud4, a known septin interactor (Kang et al., 2013), were identified both in S-phase and anaphase. The kinases Hsl1, Mck1, and Prk1 are specifically associated with the septins in S-phase cells. Ste20 and yeast SUMO (Smt3) were only identified as specific septin interactors in cells with split septin rings. An activity of Mck1 and Prk1 on the septins has not been reported and remains to be confirmed by supplementary studies. This screen strengthened also evidence that septins play a role in endocytosis: Syp1 is a negative regulator of the WASP-Arp23 complex and involved in endocytic site formation, but was also classified as septin organizing protein (Qiu et al., 2008; Merlini et al., 2015). It interacts with the septins in anaphase (Renz et al., 2016). In S-phase, the septins interact with Sla2, an adaptor protein that links actin to clathrin and endocytosis (Skruzny et al., 2015). In alpha facor arrested cells, Vps1 interacts with the septins (Renz et al., 2016). Vps1 is a dynamin related protein that functions at several stages of membrane trafficking including endocytic scission (Smaczynska-de Rooij et al., 2010, 2015).

The septins influence the composition of the ER at the bud neck and thereby render diffusion of ER membrane proteins from mother to bud. Septins, septin dependent kinases, the membrane protein Bud6 and sphingolipids were shown to be required for the ER diffusion barrier (Luedeke et al., 2005; Clay et al., 2014). Interestingly, the published septin interactome contains a hit for Erp1 (Renz et al., 2016), a member of the p24 complex that was reported to be involved in ER retention and ER-Golgi transport (Copic et al., 2009). Another constituent of COPII vesicles that interacts with p24 family members, Sfb3/Lpt1 (Manzano-Lopez et al., 2015), was additionally identified in the MS screen. These findings support the previsously published model of a septin dependent ER diffusion barrier.

Recapitulatory, the past few years have witnessed a considerable increase of published results concerning yeast septins, the majority of these in budding yeast. Efforts were made in all areas of septin biology, from structural investigations using EM, light microscopy and X-ray diffraction to reconstruction of septin assembly in complex *in vitro* systems and to elucidation of septin related processes in the living cell. All these studies together establish and/or approve the budding yeast as a prime model organism for septin research. While structural information of several of the human septin subunits is now avaliable (the PDB provides crystal structures for SEPT2, SEPT7, SEPT9, SEPT3 and the septin trimer 2/6/7), only one structure from a yeast septin was solved (Cdc11). Structure determination, the evaluation of the role of posttranslational modifications of the septins and the uncovering of so far unknown septin related processes in the living cell will represent the challenges for septin biologists for the next decade.

## Author contributions

TG and OG wrote the manuscript. OG prepared the figures.

## Funding

OG was supported by Deutsche Forschungsgemeinschaft grant JO 187/8-1.

### Conflict of interest statement

The authors declare that the research was conducted in the absence of any commercial or financial relationships that could be construed as a potential conflict of interest.
